# Fractional Wavelet-Based Generative Scattering Networks

**DOI:** 10.3389/fnbot.2021.752752

**Published:** 2021-10-26

**Authors:** Jiasong Wu, Xiang Qiu, Jing Zhang, Fuzhi Wu, Youyong Kong, Guanyu Yang, Lotfi Senhadji, Huazhong Shu

**Affiliations:** ^1^Laboratory of Image Science and Technology, Key Laboratory of Computer Network and Information Integration, Southeast University, Ministry of Education, Nanjing, China; ^2^Jiangsu Provincial Joint International Research Laboratory of Medical Information Processing, School of Computer Science and Engineering, Southeast University, Nanjing, China; ^3^Univ Rennes, INSERM, LTSI-UMR 1099, Rennes, France; ^4^Centre de Recherche en Information Biomédicale Sino-Français (CRIBs), Univ Rennes, INSERM, Rennes, France

**Keywords:** generative model, fractional wavelet scattering network, image generation, image fusion, feature-map fusion

## Abstract

Generative adversarial networks and variational autoencoders (VAEs) provide impressive image generation from Gaussian white noise, but both are difficult to train, since they need a generator (or encoder) and a discriminator (or decoder) to be trained simultaneously, which can easily lead to unstable training. To solve or alleviate these synchronous training problems of generative adversarial networks (GANs) and VAEs, researchers recently proposed generative scattering networks (GSNs), which use wavelet scattering networks (ScatNets) as the encoder to obtain features (or ScatNet embeddings) and convolutional neural networks (CNNs) as the decoder to generate an image. The advantage of GSNs is that the parameters of ScatNets do not need to be learned, while the disadvantage of GSNs is that their ability to obtain representations of ScatNets is slightly weaker than that of CNNs. In addition, the dimensionality reduction method of principal component analysis (PCA) can easily lead to overfitting in the training of GSNs and, therefore, affect the quality of generated images in the testing process. To further improve the quality of generated images while keeping the advantages of GSNs, this study proposes generative fractional scattering networks (GFRSNs), which use more expressive fractional wavelet scattering networks (FrScatNets), instead of ScatNets as the encoder to obtain features (or FrScatNet embeddings) and use similar CNNs of GSNs as the decoder to generate an image. Additionally, this study develops a new dimensionality reduction method named feature-map fusion (FMF) instead of performing PCA to better retain the information of FrScatNets,; it also discusses the effect of image fusion on the quality of the generated image. The experimental results obtained on the CIFAR-10 and CelebA datasets show that the proposed GFRSNs can lead to better generated images than the original GSNs on testing datasets. The experimental results of the proposed GFRSNs with deep convolutional GAN (DCGAN), progressive GAN (PGAN), and CycleGAN are also given.

## Introduction

Generative models have recently attracted the attention of many researchers, and they are widely used in image synthesis, image restoration, image inpainting, image reconstruction, and other applications. Many generative models have been proposed in the literature. They can be roughly classified into two types (Goodfellow et al., 2014): explicit density and implicit density models.

Among explicit density generative models, variational auto-encoders (VAEs) (Kingma and Welling, 2014) and their variants (Rezende et al., 2014; Salimans et al., 2015; Gregor et al., 2018) are most likely the most commonly used models, since they have useful latent representation, which can be used in inference queries. Kingma and Welling (2014) were the first to propose VAEs, which train an encoder and decoder simultaneously and can perform efficient inference and learning in directed probabilistic models and in the presence of continuous latent variables with intractable posterior distributions. Salimans et al. (2015) bridged the gap between Markov chain Monte Carlo (MCMC) and VAEs, and incorporated one or more steps of MCMC into variational approximation. Sohn et al. (2015) proposed a conditional VAE (CVAE), which joins existing label information in training to generate corresponding category data. Rezende and Mohamed (2015) introduced a new approach for specifying flexible, arbitrarily complex, and scalable approximate posterior distributions and made a clear improvement in the performance and applicability of variational inference. Sønderby et al. (2016) presented a ladder variational autoencoder, which uses a process similar to a ladder network and recursively corrects the generation distribution based on a data-independent approximate likelihood. Higgins et al. (2017) presented a β-VAE, which is a modification of a variational autoencoder (VAE), with special emphasis on discovering disentangled latent factors. Oord et al. (2017) proposed a simple yet powerful generative model that learns discrete representations and allowed the model to circumvent issues of posterior collapse. Gregor et al. (2018) proposed temporal difference VAE (TD-VAE), which is a generative sequence model that learns representations containing explicit beliefs about states in several steps into the future Razavi et al. (2019) proposed vector quantized variational autoencoder (VQ-VAE), which augments with powerful priors over latent codes and is able to generate samples with a quality that rival those of state-of-the-art GANs on multifaceted datasets, such as ImageNet. Simonovsky and Komodakis (2018) proposed Graph VAE, sidesteps the hurdles of linearization of discrete structures by outputting a probabilistic fully connected graph of a predefined maximum size directly at once. For more references on VAEs, see Blei et al. (2017).

Among implicit density generative models, generative adversarial networks (GANs) (Goodfellow et al., 2014) and their variants (Chen et al., 2016; Radford et al., 2016) are probably the most commonly used models, since they provide better generated images than other generative models. Goodfellow et al. (2014) were the first to propose GANs, which estimate generative models *via* an adversarial process, where a generative model G and a discriminative model D are trained simultaneously without the need for Markov chains or unrolled approximate inference networks during either training or the generation of samples. However, the application of GANs to real-world computer vision problems still encounters at least three significant challenges (Wang et al., 2021): (1) high-quality image generation; (2) diverse image generation; and (3) stable training. Therefore, many variants of GANs have been proposed to handle the three challenges. The variants can be roughly classified into two groups (Wang et al., 2021): architecture variant GANs and loss variant GANs.

In terms of architecture variant GANs, for example, Radford et al. (2016) proposed deep convolutional GAN (DCGAN), which uses a convolutional neural network (CNN) as the discriminator D and deploys a deconvolutional neural network architecture for G; the spatial upsampling ability of the deconvolution operation enables the generation of images with higher resolution compared with the original GANs. Mirza and Osindero (2014) proposed conditional GAN (CGAN), which imposes a condition of additional information, such as a class label, to control the process of data generation in a supervised manner. Chen et al. (2016) presented InfoGAN, which decomposes an input noise vector into a standard incompressible latent vector and another latent variable to capture salient semantic features of real samples. Karras et al. (2018) presented progressive GAN (PGAN) for generative high-resolution images using the idea of progressive neural networks (Rusu et al., 2017), which does not suffer from forgetting and is able to deploy prior knowledge *via* lateral connections to previously learned features. Karras et al. (2020a,b) proposed StyleGAN, which leads to an automatically learned, unsupervised separation of high-level attributes and stochastic variation in generated images and, thus, enables intuitive, scale-specific control of the synthesis. More recently, Hudson and Zitnick (2021) introduced the Generative Adversarial Transformer (GANformer), which is a generalization of the StyleGAN and a simple yet effective generalization of the vanilla transformer, for a visual synthesis task.

In terms of loss-variant GANs, for example, Arjovsky et al. (2017) proposed Wasserstein GAN (WGAN), which uses the Wasserstein distance as the loss measure for optimization instead of Kullback–Leibler divergence. Gulrajani et al. (2017) proposed an improved method for training the discriminator for a WGAN, by penalizing the norm of discriminator gradients with respect to data samples during training rather than performing parameter clipping. Nowozin et al. (2016) proposed an alternative cost, which is a function of the f-divergence, for updating the generator, which is less likely to saturate at the beginning of training. Zhu et al. (2017) proposed CycleGAN for the task of image-to-image translation. Qi (2020) presented loss-sensitive GAN (LS-GAN), which trains the generator to produce realistic samples by minimizing the designated margins between real and generated samples. Miyato et al. (2018) proposed spectral normalization GAN (SN-GAN), which uses a weight normalization technique to train the discriminator more stably. Brock et al. (2019) proposed BigGAN, which uses hinge loss instead of Jensen–Shannon divergence and a large-scale dataset to train the generator to produce more realistic samples.

Although GANs and VAEs are great generative models, they raise many questions. A significant disadvantage of VAEs is that the resulting generative models produce blurred images compared with GANs, since the quality of VAEs crucially relies on the expressiveness of their inference models. A significant disadvantage of GANs is that the training process is very difficult and may lead to unstable training and model collapse. To design a network that can maintain the characteristics of high-quality generated images of GANs as much as possible while reducing the training difficulty of GANs, Angles and Mallat (2018) proposed generative scattering networks (GSNs), which use wavelet scattering networks (ScatNets) (Bruna and Mallat, 2013) as the encoder to obtain features (or ScatNet embeddings) and the deconvolutional neural network of DCGAN (Radford et al., 2016) as the decoder to generate an image. The advantage of GSNs is that there is no need to learn the parameters of ScatNets; therefore, the difficulty of training is reduced when compared with DCGAN, while the disadvantage of GSNs is that generated images can lose details, which affects the quality of the generated images. After careful inspection, we determined that the sources of relatively low-quality generated images of GSNs include at least two aspects: (1) the expression ability of ScatNets is slightly weaker than that of CNNs used in DCGAN; (2) applying PCA (Abdi and Williams, 2010) to reduce the dimension of the feature map of ScatNets in the encoder part of GSNs leads to an overfitting problem in the testing process of GSNs. This finding leads to the central question of our study:

Can we change the feature extraction method of ScatNets to a more powerful one that still does not need learning? Can we develop a more suitable dimensionality reduction method to solve the overfitting problem in the testing process of GSNs?

In an attempt to solve the above questions, in this study, we propose generative fractional scattering networks (GFRSNs), which can be seen as an extension of GSNs. The contributions of this article are as follows:

1) We use, for more expressiveness, fractional wavelet scattering networks (FrScatNets) (Liu et al., 2019) instead of ScatNets (Bruna and Mallat, 2013) to extract features of images, and we use image fusion (Liu et al., 2016; Yang et al., 2017) in GFRSNs to effectively improve the visual quality of the generated images.
2) We propose a new dimensionality reduction method named feature-map fusion (FMF), which is more suitable for reducing the feature dimension of FrScatNets than PCA, since the FMF method greatly alleviates the overfitting problem on the testing datasets using GFRSNs.
3) The image generated by the proposed GFRSN on the test set is better than that produced by the original GSNs.

The remainder of this article is organized as follows: In section Generative Scattering Networks (GSNs), wavelet scattering networks and the architectural components of GSNs are briefly introduced. The main architectural components of GFRSNs, which include fractional wavelet scattering networks, the FMF dimensionality reduction method and generative networks, and an image fusion method are introduced in section Generative Fractional Scattering Networks (GFRSNs). The performance of GFRSNs is analyzed and compared with that of the original GSNs in section Numerical Experiments. The conclusions and further discussion are presented in section Conclusions.

## Generative Scattering Networks (GSNS)

**Figure 1:**
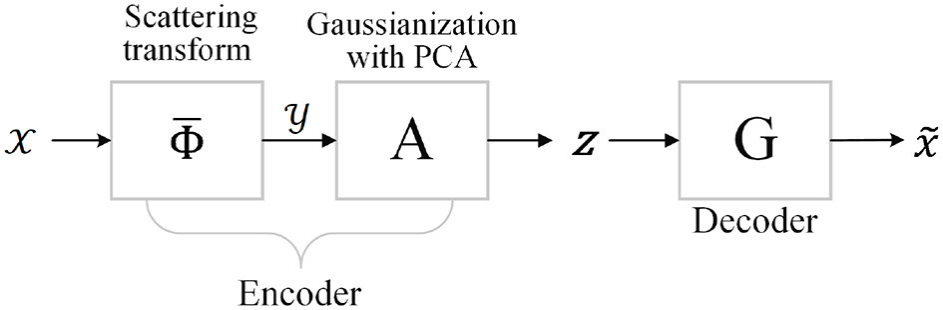
Structure of generative scattering networks (GSNs) with principal component analysis (PCA) dimensionality reduction method.

In this section, we first briefly recall the generative scattering networks (GSNs) (Angles and Mallat, 2018), whose structure is shown in Figure 1.

The input *M*th-order tensor $X\in {\mathbb{R}}^{N_{1}\times N_{2}\times \cdots \times N_{K}}$, where ℝ denotes the real domain and each *N*_*i*_,*i* = 1, 2, 3, ⋯*K*, addresses the i-mode of X, and is first fed into the feature extraction part of the encoder to obtain the ScatNet features $Y\in {\mathbb{R}}^{M_{1}\times M_{2}\times \cdots \times M_{L}}$. The next part of the encoder aims to map the features to a Gaussian latent variable **z**∈ℝ^*U*^, which is accomplished by whitening and projection to a lower-dimensional space. Inspired by Zou and Lerman (2019), we refer to this process as Gaussianization. Decoder G can be seen as a generator and is trained by minimizing the reconstruction loss between the output $\tilde{X}\in {\mathbb{R}}^{N_{1}\times N_{2}\times \cdots \times N_{K}\;}$ and input X.    In other words, the generator calculation is regarded as the inverse problem of the scattering transform. The main components of GSNs include ScatNets, Gaussianization with PCA, and the generative network G. These components are recalled as follows.

### Wavelet Scattering Networks (ScatNets)

Let the complex bandpass filter ψ_λ_ be constructed by scaling and rotating a filter ψ, respectively, by 2^*j*^ and δ, as follows (Bruna and Mallat, 2013):


(1) $$\begin{array}{l} \psi_{\lambda }\left(t\right)=2^{2j}\psi \left(2^{j}\delta^{-1}t\right),\lambda =2^{j}\delta , \end{array}$$

with 0 ≤ *j* ≤ *J*−1, and δ = *kπ*/*K, k* = 0, 1, ..., *K*−1.

The wavelet-modulus coefficients of *x* are given by:


(2) $$\begin{array}{l} U\left[\lambda \right]x=|x\;*\;\psi_{\lambda }|. \end{array}$$

The scattering propagator *U*[*p*] is defined by cascading wavelet-modulus operators


(3) $$\begin{array}{l} U\left[p\right]x=U\left[\lambda_{m}\right]\cdots U\left[\lambda_{2}\right]U\left[\lambda_{1}\right]x\\ \;=\text{|}\text{|}|x*\psi_{\lambda_{1}}|*\psi_{\lambda_{2}}\text{|}\cdots *\psi_{\lambda_{m}}\text{|}, \end{array}$$

where *p* = (λ_1_, λ_2_, .., λ_*m*_) are the frequency-decreasing paths; in other words, |λ_*k*_| ≥ |λ_*k*+1_|, *k* = 1, 2, ..., *m* − 1. Note that *U*[∅]*x* = *x*, and ∅ expresses the empty set.

The scattering operator *S*_*J*_ performs spatial averaging on a domain whose width is proportional to 2^*J*^:


(4) $$\begin{array}{l} S\left[p\right]x=U\left[p\right]x*\phi_{J}=U\left[\lambda_{m}\right]\cdots U\left[\lambda_{2}\right]U\left[\lambda_{1}\right]x*\phi_{J}\\ \;=\text{|}\text{|}|x*\psi_{\lambda_{1}}|*\psi_{\lambda_{2}}\text{|}\cdots *\psi_{\lambda_{m}}\text{|}*\phi_{J}. \end{array}$$

The network nodes of layer m correspond to the set *P*^*m*^of all paths *p* = (λ_1_, λ_2_, .., λ_*m*_) of length *m*. This *m*-th layer stores the propagated signals${\left\{U\left[p\right]x\right\}}_{p\in P^{m}}$and outputs the scattering coefficients ${\left\{S\left[p\right]x\right\}}_{p\in P^{m}}$. The output is obtained by cascading the scattering coefficients of every layer.

Note that *x* in (2) can be one-dimensional data $\text{x}\in {\mathbb{R}}^{N_{1}}$, two-dimensional data $\text{X}\in {\mathbb{R}}^{N_{1}\times N_{2}}$, and third-order tensor $X\in {\mathbb{R}}^{N_{1}\times N_{2}\times N_{3}}$, which can be seen as *N*_3_ two-dimensional data $\text{X}\in {\mathbb{R}}^{N_{1}\times N_{2}}$, and ScatNet addresses with these ***X*_*s*_** one by one and then superimposes the results as output features. According to Mallat (2012), if we feed the input $X\in {\mathbb{R}}^{N_{1}\times N_{2}\times N_{3}}\;$ into ScatNet, then we can obtain ScatNet features (or ScatNet embeddings) as follows:


(5) $$\begin{array}{l} Y=S\left[p\right]X\in {\mathbb{R}}^{N_{3}\times \left(1+LJ+L^{2}J\left(J-1\right)/2\right)\times \left(N_{1}/2^{J}\right)\times \left(N_{2}/2^{J}\right)}, \end{array}$$

where *N*_3_ is the number of input sample channels, and *N*_1_ and *N*_2_ are the width and height of the input sample, respectively. *N*_1_/2^*J*^ and *N*_2_/2^*J*^ are the width and height of the output features. *J* is a scale factor, and *L* is the number of rotation angles. Note that the number of feature maps in the first, second, and third layers is 1, *LJ*, and *L*^2^*J*(*J*-1)/2, respectively.

### Gaussianization With PCA

As shown in Figure 1, the last step of the encoder maps the transformed features in such a way that we can sample from the Gaussian distribution to generate new images, as required by the generator. Specifically, let ${\left\{Y\right\}}_{t=1}^{T}$ be the output features of the ScatNet embedding, and let Y be the representing matrix of ${\left\{Y\right\}}_{t=1}^{T},$ while **z** is the latent variable of the generator. As advocated in Angles and Mallat (2018), **z** can be interpreted as an address, with a dimension *d* lower than that in the input image. Hence, to get a lower-dimensional embedding of the output features, one can perform principal component analysis (PCA) (Abdi and Williams, 2010) to project the features of the scattering transform to a lower-dimensional space.

Next, to whiten them, we choose $u=\frac{1}{T}\sum_{t=1}^{T}Y,\sum =\frac{1}{T}\sum_{t=1}^{T}\left(Y-u\right){\left(Y-u\right)}^{*},$ and the whitening map *A* = ∑^−1/2^(*Id*−*u*).

Hence, the resulting embedding of the encoder is


(6) $$\begin{array}{l} \text{z}=\sum^{-1/2}\left(Y-u\right). \end{array}$$

After the above process, the whitened sample is uncorrelated, and their distribution will be close to a normal one with identity covariance (Angles and Mallat, 2018), which is exactly what we want to feed to the generator.

### Generator Networks in GSNs

The generative network G of GSNs is a neural one, which is similar to the generator of DCGAN (Radford et al., 2016), which inverts the whitened scattering embedding on training samples. The generator network G includes a fully connected layer (FC), batch normalization layer (BN) (Ioffe and Szegedy, 2015), bilinear upsampling (Upsample) layer, and convolutional layer (Conv2d) with a kernel size of 7 × 7. Except for the last layer, which uses the tanh activation function, the others use the default ReLU (Nair and Hinton, 2010) activation function.

Generative scattering networks with PCA as the dimensionality reductional method choose the *L*_1_-norm loss function and solve the following optimization problem (Zhao et al., 2017):


(7) $$\begin{array}{l} g_{1}=min\;Loss_{L_{1}}\left(X,\tilde{X}\right)=min\;\frac{1}{N}\overset{N}{\underset{i=1}{\sum }}|X^{\left(i\right)}-{\tilde{X}}^{\left(i\right)}|, \end{array}$$

where X represents the input data, $\tilde{X}$ represents the generative data, $X^{\left(i\right)}$ represents the *i*-th input sample, and ${\tilde{X}}^{\left(i\right)}\;$ represents the *i*-th generative sample:


(8) $$\begin{array}{l} \tilde{X}=G\left(PCA\left(S\left[p\right]X\right)\right), \end{array}$$

where S[p]X denotes the feature extraction process with ScatNets, and PCA(.) represents that the feature dimensionality reduction method is PCA. *G*(.) represents the generative network G. The optimization problems in (7) are then solved with the Adam optimizer (Kingma and Ba, 2015) using the default hyperparameters.

## Generative Fractional Scattering Networks (GFRSNS)

**Figure 2:**
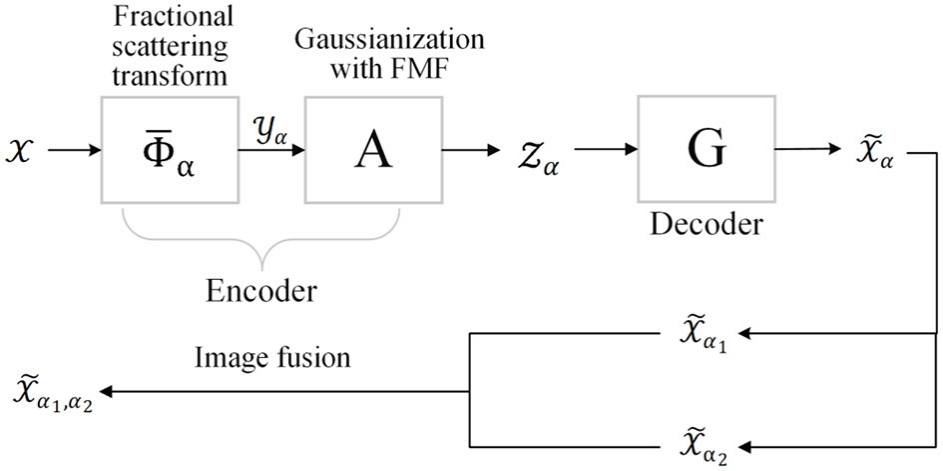
Structure of generative fractional scattering networks (GFRSNs).

In this section, we introduce the proposed generative fractional scattering networks (GFRSNs), whose structure is shown in Figure 2.

The input $X\in {\mathbb{R}}^{N_{1}\times N_{2}\times \cdots \times N_{K}}$ is first fed into the fractional wavelet scattering networks (FrScatNets) to obtain FrScatNet features (or FrScatNet embeddings) $Y_{\alpha }\in {\mathbb{R}}^{M_{1}\times M_{2}\times \cdots \times M_{L}}$, whose dimensions are then reduced by the proposed feature-map fusion (FMF) method to obtain an implicit tensor $Z_{\text{α}}\in {\mathbb{R}}^{O_{1}\times O_{2}\times \cdots \times O_{K}}$, which is then fed into the generator G to obtain the generated output tensor ${\tilde{X}}_{\alpha }\in {\mathbb{R}}^{N_{1}\times N_{2}\times \cdots \times N_{K}}$. In other words, the generative network G is seen as the inverse problem of FrScatNets. The main components of GFRSNs include FrScatNets, Gaussianization with feature-map fusion dimensionality reduction method, and the generative network G. In the following, these components of GFRSNs are introduced.

### Fractional Wavelet Scattering Networks (FrScatNets)

In this subsection, fractional wavelet scattering networks (FrScatNets) (Liu et al., 2019) are briefly introduced. Similar to (2), the fractional wavelet modulus coefficients of *x* are given by:


(9) $$\begin{array}{l} U_{\alpha }\left[\lambda \right]x=|x\Theta_{\alpha }\psi_{\lambda }|, \end{array}$$

where Θ_α_ is the fractional convolution defined by Shi et al. (2010);


(10) $$\begin{array}{l} x\left(t\right)\Theta_{\alpha }\psi_{\lambda }\left(t\right)=\;e^{-\frac{j}{2}t^{2}\cot\theta }\left[\left(x\left(t\right)e^{\frac{j}{2}t^{2}\cot\theta }\right)\text{*}\psi_{\lambda }\left(t\right)\right], \end{array}$$

where the parameter α is the fractional order and θ = απ*/2* represents the rotation angle. Note that when α = 1, the fractional convolution in (10) reduces to conventional convolution in (2).

The fractional scattering propagator *U*_α_[*p*] is defined by cascading fractional wavelet modulus operators


(11) $$\begin{array}{l} U_{\alpha }\left[p\right]x=U_{\alpha }\left[\lambda_{m}\right]\cdots U_{\alpha }\left[\lambda_{2}\right]U_{\alpha }\left[\lambda_{1}\right]x\\ \;=\text{|}\text{|}|x\Theta_{\alpha }\psi_{\lambda_{1}}|\Theta_{\alpha }\psi_{\lambda_{2}}\text{|}\cdots \Theta_{\alpha }\psi_{\lambda_{m}}\text{|}, \end{array}$$

where *p* = (λ_1_, λ_2_, .., λ_*m*_)are the frequency-decreasing paths; in other words, |λ_*k*_| ≥ |λ_*k* + 1_|, *k* = 1, 2, ..., *m* − 1. Note that *U*_α_[∅]*x* = *x*, and ∅ expresses the empty set.

The fractional scattering operator *S*_α_ performs spatial averaging on a domain whose width is proportional to 2^*J*^:


(12) $$\begin{array}{l} S_{\alpha }\left[p\right]x=U_{\alpha }\left[p\right]x\Theta_{\alpha }\phi_{J}=U_{\alpha }\left[\lambda_{m}\right]\cdots U_{\alpha }\left[\lambda_{1}\right]x\Theta_{\alpha }\phi_{J}\\ \;=\text{|}\text{|}|x\Theta_{\alpha }\psi_{\lambda_{1}}|\Theta_{\alpha }\psi_{\lambda_{2}}\text{|}\cdots \Theta_{\alpha }\psi_{\lambda_{m}}\text{|}\Theta_{\alpha }\phi_{J}. \end{array}$$

**Figure 3:**
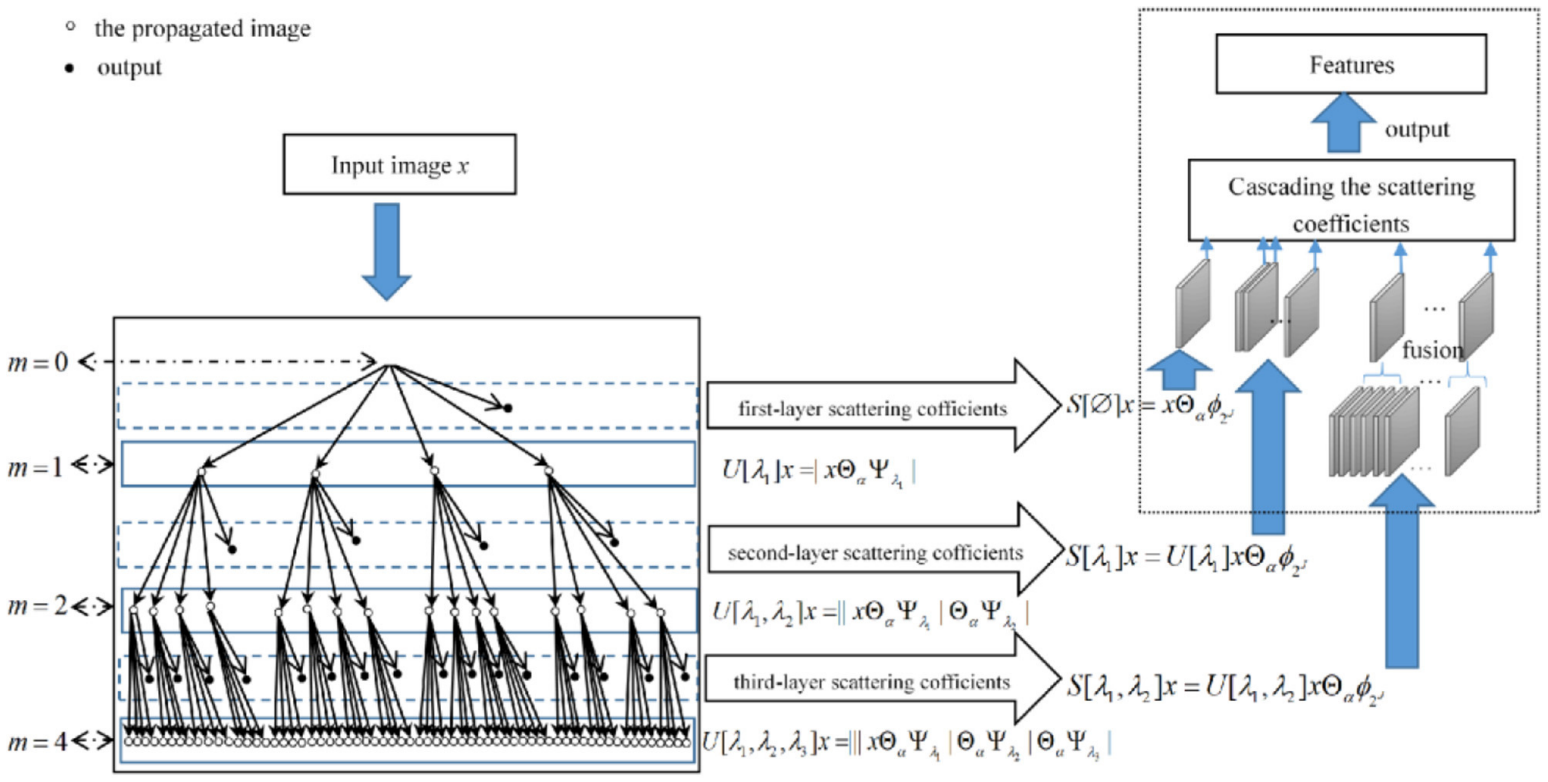
Fractional wavelet scattering network and the feature-map fusion dimensional reduction method.

The structure of FrScatNets is shown on the left of Figure 3.

The network nodes of the layer m correspond to the set *P*^*m*^ of all paths *p* = (λ_1_, λ_2_, .., λ_*m*_) of length *m*. This *m*-th layer stores the propagated signals $\;{\left\{U_{\alpha }\left[p\right]x\right\}}_{p\in P^{m}}$ and outputs the fractional scattering coefficients$\;{\left\{S_{\alpha }\left[p\right]x\right\}}_{p\in P^{m}}$. The output is obtained by cascading the fractional scattering coefficients of every layer. Note that when α = 1, the FrScatNets in (12) default to conventional ScatNets in (4), since the fractional convolution in (10) reduces to conventional convolution in (2).

Note that FrScatNets retain the advantages of ScatNets, for example, no need for learning, translation-invariant property, linearized deformations, and certain parameters. Compared with ScatNets, FrScatNet adds a free parameter α, which represents fractional order. With α continuously growing from 0 to 2, FrScatNets can show the characteristics of an image from time domain to frequency domain. Thus, FrScatNets provide more fractional domain choices for the feature extraction of input data. Furthermore, for the image generation task in this study, we can obtain as many generated images from FrScatNet embeddings as different fractional orders α_*i*_, and then they can be fused to further improve the quality of the generated images.

If we feed the input $X\in {\mathbb{R}}^{N_{1}\times N_{2}\times N_{3}}$ into the FrScatNet, then we can obtain the features of FrScatNet (or FrScatNet embeddings) as follows:


(13) $$\begin{array}{l} Y_{\alpha }=S_{\alpha }\left[p\right]X\in {\mathbb{R}}^{N_{3}\times \left(1+LJ+L^{2}J\left(J-1\right)/2\right)\times \left(N_{1}/2^{J}\right)\times \left(N_{2}/2^{J}\right)}. \end{array}$$

Note that the size of output features of FrScatNets is the same as that of ScatNets, whose size is shown in (5).

### Gaussianization With FMF

In this subsection, we introduce a new method called FMF to reduce the dimensionality of the features after a fractional scattering transformation. We propose such an algorithm based on the hierarchical tree structure of features extracted by the fractional scattering transform to replace PCA to map the features to a low-dimensional space. More specifically, since the output features of different layers from the fractional scattering transform have a hierarchical structure, which is not considered in the PCA algorithm, we need a dimensionality reduction method that can make full use of this hierarchical information. The number of feature maps in the first, second, and third layers is 1, *LJ*, and *L*^2^*J(J-1)/2*, respectively. Obviously, the third layer has the largest number of feature maps. Therefore, we fuse only the feature maps from the third layer of the fractional scattering transform to significantly reduce the data dimension. The fusion method is very simple: we obtain a new feature map by simply taking the average of every *L(J-1)/2* feature map, which obtains *LJ* feature maps after applying the FMF method to the output of the third layer of FrScatNets. The dotted box on Figure 3 illustrates the proposed FMF method.

Therefore, an input tensor $X\in {\mathbb{R}}^{N_{1}\times N_{2}\times N_{3}}$is fed into the FrScatNets to obtain FrScatNet features $Y_{\text{α}}$ in (13), which are then processed by the FMF method, obtaining an implicit tensor


(14) $$\begin{array}{l} Z_{\alpha }=FMF\left(Y_{\alpha }\right)\in {\mathbb{R}}^{N_{3}\times \left(1+LJ+LJ\right)\times \left(N_{1}/2^{J}\right)\times \left(N_{2}/2^{J}\right)}, \end{array}$$

whose size is significantly smaller than the size shown in (13) without using the FMF method. Note that FMF(.) means performing the FMF method.

The obtained implicit tensor $Z_{\text{α}}$ is then input to the generator network G, described below, to obtain the generated image.

### Generative Networks in GFRSNs

The generative network G of GFRSNs is also a deconvolutional neural network that has a generator similar to that of DCGAN (Radford et al., 2016), which inverts fractional scattering embeddings on training samples. The generative network G of GFRSNs also includes a fully convolutional layer (Fully Conv) (Long et al., 2015) and several convolution blocks that consist of bilinear upsampling (UP), two convolutional layers (Conv) with a 3 × 3 kernel size, batch normalization, and ReLU (the activation function of the last convolution layer is tanh). GFRSNs also choose the *L*_1_-norm loss function and solve the following optimization problem:


(15) $$\begin{array}{l} g_{2}=\min Loss_{L_{1}}\left(X,{\tilde{X}}_{\alpha }\right)=\min\frac{1}{N}\overset{N}{\underset{i=1}{\sum }}|X^{\left(i\right)}-{\tilde{X}}_{\alpha }^{\left(i\right)}|, \end{array}$$

where ${\tilde{X}}_{\alpha }$ represents the generative data and ${\tilde{X}}_{\alpha }^{\left(i\right)}$. represents the *i*-th generative sample, and


(16) $$\begin{array}{l} {\tilde{X}}_{\alpha }=G\left(FMF\left(S_{\alpha }\left[p\right]X\right)\right), \end{array}$$

where $S_{\alpha }\left[p\right]X$ denotes the feature extraction process with FrScatNets, FMF(.) represents the dimensionality reduction process, and *G*(.) represents the generative network.

The optimization problem in (15) is then solved with the Adam optimizer (Kingma and Ba, 2015).

### Image Fusion

In contrast to GSNs, the proposed generative fractional scattering networks (GFRSNs) embed the input using FrScatNets, which allows for deriving many embeddings, since FrScatNets have an additional fractional order α; therefore, we can embed the input in different fractional order domains. These FrScatNet embeddings may extract many different but complementary features from the input. We can effectively use these embeddings to generate many images and further improve the quality of the synthesized images using fusion methods. In this study, as shown at the bottom of Figure 2, we use a simple image fusion method that is weighted average. As examples, we simply use the following:


(17) $$\begin{array}{l} {\tilde{X}}_{\alpha_{1},\alpha_{2}}=\lambda {\tilde{X}}_{\alpha_{1}}+\left(1-\lambda \right){\tilde{X}}_{\alpha_{2}}, \end{array}$$

where λ is the balanced parameter, which is set here to 0.5.

## Numerical Experiments

In this section, we evaluate the quality of the generated images by the proposed GFRSNs by means of several experiments. The quality of the generated images is evaluated with two criteria: peak signal to noise ratio (PSNR) (Wang et al., 2003) and structural similarity (SSIM) (Wang et al., 2004).

We performed experiments on two datasets that have different levels of variability: CIFAR-10 (Krizhevsky, 2009) and CelebA (Liu et al., 2015). The CIFAR-10 dataset includes 50,000 training images and 10,000 testing images, whose sizes are 32 × 32 × 3. In all the experiments on the CIFAR-10 dataset, after image grayscale preprocessing, the number of rotation angles *L* is set to 8, and the fractional scattering averaging scale is set to 2^*J*^ = 2^3^ = 8, which means that we linearize translations and deformations of up to 8 pixels. Therefore, the size of the output features from FrScatNets according to Equation (13) is 1 × 217 × 4 × 4, which is then, after the FMF method according to Equation (14), reduced to 1 × 49 × 4 × 4 (the size of implicit tensor $Z_{\text{α}}$). In addition, the CelebA dataset contains thousands of images, and we choose 65,536 training images and 16,384 testing images, whose sizes are 128 × 12 8 × 3. In all the experiments on the CelebA dataset, after image grayscale preprocessing, the number of rotation angle *L* is set to 8, and the fractional scattering averaging scale is set to 2^*J*^ = 2^4^ = 16, which means that we linearize translations and deformations of up to 16 pixels. Thus, the size of the output features from FrScatNets according to (13) is 1 × 417 × 8 × 8, which is then, after FMF method according to Equation (14), reduced to 1 × 65 × 8 × 8 (the size of implicit tensor $Z_{\text{α}}$). Table 1 shows the core parameters of FrScatNet and its settings on the CIFAR-10 and CelebA datasets.

**Table 1:** Core parameters of FrScatNet with and without feature dimensionality reduction.

**Parameter**	**Descriptions**	**Dataset**	
		**CIFAR-10**	**CelebA**
*N*_1_ × *N*_2_ × *N*_3_	The size of input image	32 × 32 × 1	128 × 128 × 1
*J*	The fractional scattering averaging scale	3	4
*L*	The number of rotation angle	8	8
$N_{3}\times \left(1+LJ+\frac{L^{2}J\left(J-1\right)}{2}\right)\times \frac{N_{1}}{2^{J}}\times \frac{N_{2}}{2^{J}}$	The shape of FrScatNets features $Y_{\text{α}}$	1 × 217 × 4 × 4	1 × 417 × 8 × 8
$N_{3}\times \left(1+2\times LJ\right)\times \frac{N_{1}}{2^{J}}\times \frac{N_{2}}{2^{J}}$	The shape of implicit tensor $Z_{\text{α}}$ with FMF	1 × 49 × 4 × 4	1 × 65 × 8 × 8

In the following, we first compare the visual quality of the generated images with different feature dimensionality reduction methods in the framework of GFRSNs. Then, we compare the visual quality of the generated images with FrScatNets. Finally, we compare the visual quality of the fused images and unfused images. The following experiments are implemented using PyTorch on a PC machine, which sets up an Ubuntu 16.04 operating system and has an Intel (R) Core(TM) i7-8700K CPU with a speed of 3.7 GHz and 32 GB RAM, and has two NVIDIA GeForce GTX1080-Ti GPUs.

### Image Generative Results With Different Dimensionality Reduction Methods

In this subsection, we compare the results on the quality of generative images with two different dimensionality reduction methods: the PCA method and the proposed FMF method. We set the fractional orders to be α_1_ = α_2_ = 1, and use conventional ScatNets to extract features from the input X for simplicity.

For the PCA-based GFRSNs, the flow chart is shown in Figure 1. For the CIFAR-10 dataset, the size of the implicit vector **z** is 49 × 4 × 4 = 784, and for the CelebA dataset, the size of the implicit vector **z** is 65 × 8 × 8 = 4,160. We use the PyTorch code of generative scattering networks^1^ provided by Tomás Angles. The PSNR and SSIM on the CIFAR-10 and CelebA datasets are shown in the second columns of Tables 2, 3, respectively.

**Table 2:** Peak signal to noise ratio (PSNR) and structural similarity (SSIM) scores of training and testing images from FrScatNets with fractional orders α_1_ = α_2_ = 1 on the CIFAR-10 dataset.

	**PCA**	**Feature-Map Fusion**	**Increased (%)**
Train PSNR	**23.08**	20.1500	−12.69
Test PSNR	17.92	**18.1000**	**1.00**
Train SSIM	**0.9428**	0.8859	−6.08
Test SSIM	0.8206	**0.8352**	**1.78**

*Increased means the percentages of relative improvements of FMF over principal component analysis (PCA), the better results are shown in bold*.

**Table 3:** PSNR and SSIM scores of training and testing images from FrScatNets with fractional orders α_1_ = α_2_ = 1 on the CelebA dataset.

	**PCA**	**Feature-Map Fusion**	**Increased (%)**
Train PSNR	**23.8124**	22.7526	−4.45
Test PSNR	**20.5312**	19.7688	−3.71
Train SSIM	**0.9529**	0.944	−0.93
Test SSIM	**0.9104**	0.8993	−1.22

*Increased means the percentage of relative improvements of FMF over PCA, the better results are shown in bold*.

As shown in the two tables, the scores of PSNR (Train PSNR) and SSIM (Train SSIM), both in the training dataset, are very good for the PCA-based GFRSNs; however, their corresponding values (test PSNR and test SSIM) in the testing dataset are slightly low. This phenomenon indicates that an overfitting problem has occurred using the PCA-based GFRSNs. We argue the reason behind this phenomenon could be that the output feature of FrScatNets $Y_{\text{α}}$ in (16) is a 4th-order tensor, which is performed by PCA to obtain an implicit vector **z**. This process loses correlations between various dimensions of the data. Therefore, we consider using FMF as the dimensionality reduction method to maintain the structures of the input data better.

For the proposed FMF-based GFRSNs, the flow chart is shown in Figure 2. The size of the implicit tensor Z_α_*i*__ is 1 × 49 × 4 × 4 on CIFAR-10, and for the CelebA dataset, the size of implicit tensor Z_α_*i*__ is 1 × 65 × 8 × 8. The PSNR and SSIM on the CIFAR-10 and CelebA datasets are shown in the third columns of Tables 2, 3, respectively. As can be seen from the two tables, train PSNR and train SSIM of the FMF-based GFRSNs are slightly worse than those of the PCA-based GFRSNs on the CIFAR-10 and CelebA datasets; however, the test PSNR and test SSIM of the proposed FMF-based GFRSNs are better than those of the PCA-based GFRSNs. For example, Test PSNR and Test SSIM have relatively increased by 1 and 1.8%, respectively, when compared with the PCA-based GFRSNs, on the CIFAR-10 dataset. However, with regard to the CelebA dataset, Test PSNR and Test SSIM have decreased by 3.71 and 1.22%, respectively, when compared with the PCA-based GFRSNs. Nevertheless, the experimental results still show that the overfitting problem on the testing datasets can be alleviated with the FMF dimensionality reduction method.

Although the performance of the proposed FMF method on theCIFAR-10 dataset is better than that of PCA and has a similar generation ability on the CelebA dataset, more importantly, FMF has better generalization performance under the framework of GFRSNs. In other words, our generative model will not overfit on the test set. However, in order to better reflect the role of fractional scattering transformation and, hence, abolish the influence of FMF, we still use the PCA method in the following two experiments.

### Image Generative Results With Different Fractional Order α

In this subsection, we explore the impact of fractional order α on the quality of the generated image using the framework of GFRSNs shown in Figure 1. The other parameter settings of FrScatNets are shown in Table 1. We choose the *L*_1_ loss function in (15) and train the generator with the Adam optimizer using the default hyperparameters.

In this subsection, we use a two-dimensional fractional Morlet wavelet to construct the FrScatNets. For the two-dimensional fractional wavelet, two fractional orders, α_1_ and α_2_, are needed to determine the rotational angle. The angle is defined as θ = α *π* /2, ranging from 0 to π ; thus, the fractional orders α_1_ and α_2_ change from 0 to 2. To save computation time, we fix one order as 1 and the other order changes within the range 0–2 for computing the fractional scattering coefficients. The chosen values are 0.1, 0.4, 0.7, 1, 1.3, 1.6, and 1.9. The above parameter settings are same as those in Liu et al. (2016). Note that FrScatNets reduce to conventional ScatNets when α_1_ = α_2_ = 1. The PSNR and SSIM of the generated images from FrScatNets on the CIFAR-10 and CelebA datasets are shown in Tables 4, 5.

**Table 4:** Results with FrScatNets on the CIFAR-10 dataset.

**(α_**1**_, α_**2**_)**	**Fusion**	**Test PSNR**	**Increased (%)**	**Test SSIM**	**Increased (%)**
Base line with (α_1_, α_2_) = (1.00,1.00)	No	18.1000	0	0.8352	0
(0.10,1.00)	No	13.9738	−22.80	0.5442	−34.84
	Yes	16.9597	−6.30	0.6974	−16.50
(0.40,1.00)	No	**18.8280**	4.02	**0.8514**	**1.94**
	Yes	**18.9869**	4.90	**0.8970**	**7.40**
(0.70,1.00)	No	18.6614	3.10	0.8469	1.40
	Yes	18.8421	4.10	0.8887	6.40
(1.30,1.00)	No	18.6169	2.86	0.8462	1.32
	Yes	18.8059	3.90	0.8870	6.20
(1.60,1.00)	No	**18.8209**	**3.98**	**0.8517**	**1.98**
	Yes	**18.9688**	**4.80**	**0.8987**	**7.60**
(1.90,1.00)	No	14.0110	−22.59	0.5474	−34.46
	Yes	16.9959	−6.10	0.7041	−15.70
(1.00,0.10)	No	14.0099	−22.60	0.5498	−34.17
	Yes	16.9054	−6.60	0.6941	−16.90
(1.00,0.40)	No	18.9351	4.61	0.8550	2.37
	Yes	18.9507	4.70	0.8978	7.50
(1.00,0.70)	No	18.7289	3.47	0.8499	1.76
	Yes	18.7335	3.50	0.8753	4.80
(1.00,1.30)	No	18.6947	3.29	0.8434	0.98
	Yes	18.5887	2.70	0.8753	4.80
(1.00,1.60)	No	18.9056	4.45	0.8545	2.31
	Yes	18.9507	4.70	0.8987	7.60
(1.00,1.90)	No	14.0487	−22.38	0.5520	−33.91
	Yes	16.9778	−6.20	0.7074	−15.30
Fusing (0.40,1.00) and (1.60,1.00)	Yes	**19.1589**	**5.85**	**0.8927**	**6.89**

*Some better results are shown in bold. In the second column, “No” means un-fused image, and “Yes” means fused image. We also show the percentages of relative improvements on Test PSNR and Test SSIM of FrScatNets of various fractional orders (**α**_1_, **α**_2_) over the conventional ScatNets (the first row), respectively*.

**Table 5:** Results with FrScatNets on CelebA dataset.

**(α_**1**_, α_**2**_)**	**Fusion**	**Test PSNR**	**Increased (%)**	**Test SSIM**	**Increased (%)**
Base line with (α_1_, α_2_) = (1.00,1.00)	No	21.1668	0	0.9221	0
(0.10,1.00)	No	18.3728	−13.2	0.7709	−16.4
	Yes	21.0186	−0.7	0.9156	−0.7
(0.40,1.00)	No	21.4631	**1.1**	**0.9350**	**3.3**
	Yes	22.2040	**5.3**	**0.9608**	**6.5**
(0.70,1.00)	No	21.3996	1.1	0.9525	2.3
	Yes	22.3098	5.4	0.9820	6.2
(1.30,1.00)	No	21.3785	1	0.9433	2.4
	Yes	22.3098	5.4	0.9793	6.2
(1.60,1.00)	No	**21.4631**	**1.4**	**0.9571**	**3.8**
	Yes	**22.3310**	**5.5**	**0.9839**	**6.7**
(1.90,1.00)	No	18.6268	−12	0.7866	−14.7
	Yes	21.1456	−0.1	0.9219	−0.02
(1.00,0.10)	No	18.2458	−13.8	0.7561	−18
	Yes	20.9551	−1	0.9092	−1.4
(1.00,0.40)	No	21.5055	1.6	0.9405	2
	Yes	22.3098	5.4	0.9756	5.8
(1.00,0.70)	No	21.2515	0.4	0.9249	0.3
	Yes	22.2251	5	0.9700	5.2
(1.00,1.30)	No	21.2303	0.3	0.9267	0.5
	Yes	22.2251	5	0.9581	3.9
(1.00,1.60)	No	21.4843	1.5	0.9433	2.3
	Yes	22.3098	5.4	0.9765	5.9
(1.00,1.90)	No	18.7115	−11.6	0.7912	−14.2
	Yes	21.1732	0.03	0.9212	−0.1
Fusing (0.40,1.00) and (1.60,1.00)	Yes	**22.0770**	**4.3**	**0.9802**	**6.3**

*Some better results are shown in bold. In the second column, “No” means un-fused image, and “Yes” means fused image. We also show the percentages of relative improvements on Test PSNR and Test SSIM of FrScatNets of various fractional orders (α_1_, α_2_) over the conventional ScatNets (the first row), respectively*.

Generally, as shown in Table 4, best results are not obtained using FrScatNets with (α_1_, α_2_) = (1, 1), which means that FrScatNets with some fractional order choice of (α_1_, α_2_) obtain better embeddings than the conventional ScatNets. For example, both the PSNR and SSIM results are very good the FrScatNets with (α_1_, α_2_) = (0.4, 1.00) were used and whose Test PSNR and Test SSIM increased by 4.2 and 1.9%, respectively, compared with those of the ScatNets.

For the CelebA dataset, as shown in Table 5, both the PSNR and SSIM scores in the test set are also very good when FrScatNets with (α_1_, α_2_) = (1.6, 1) are used. Indeed, Test PSNR and Test SSIM increased by 1.4 and 3.8%, respectively, compared with those of the ScatNets.

**Figure 4:**
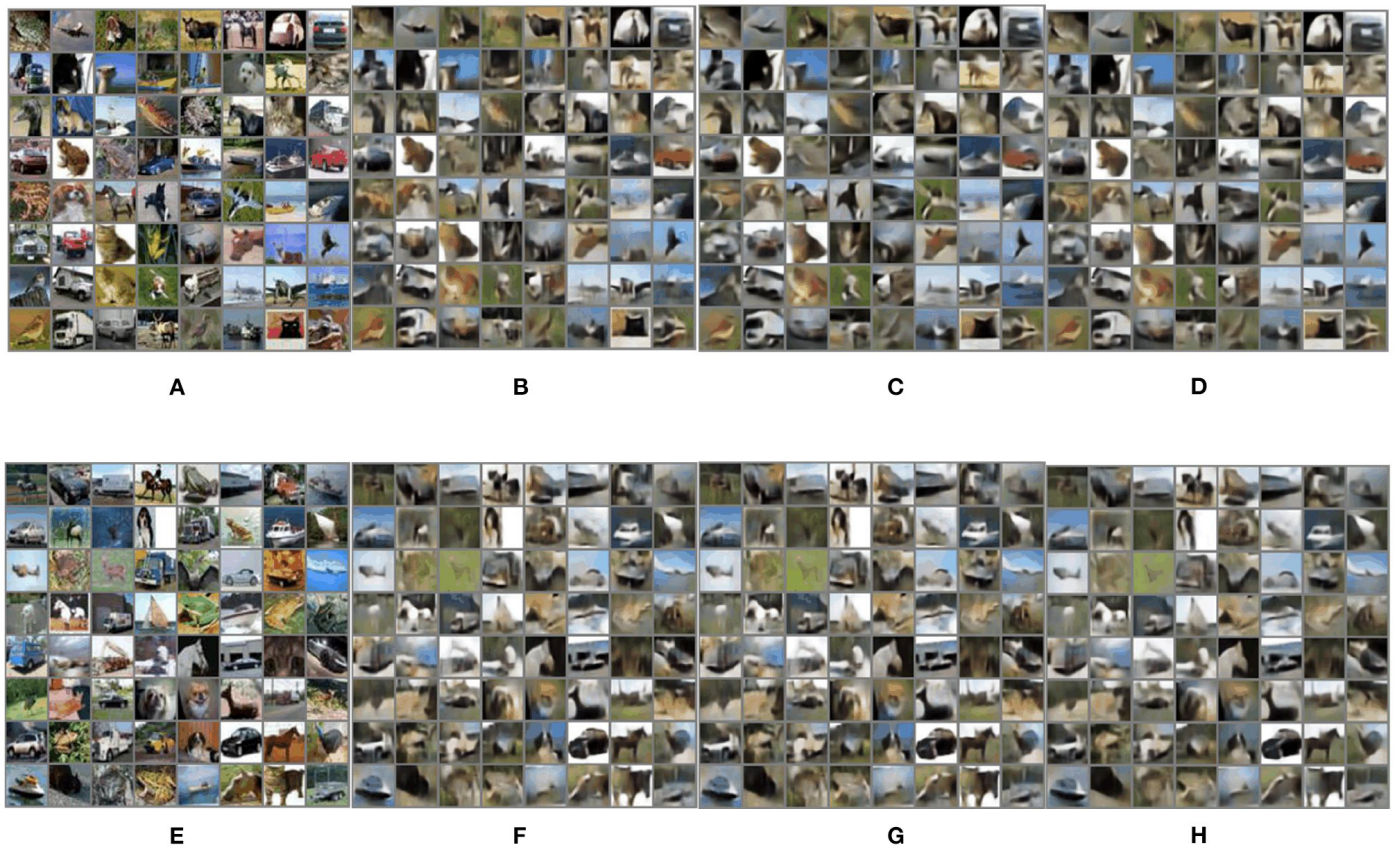
Generative images on CIFAR-10 dataset using FrScatNet embeddings. **(A)** Original training images; **(B)** generative training images using FrScatNets with (α_1_, α_2_) = (0.4, 1); **(C)** generative training images using FrScatNets with (α_1_, α_2_) = (1, 1); **(D)** fused training image using FrScatNets with (α_1_, α_2_) = (0.4, 1 and (α_1_, α_2_) = (1, 1); **(E)** original testing images; **(F)** generative testing images using FrScatNets with (α_1_, α_2_) = (0.4, 1); **(G)** generative testing images using FrScatNets with (α_1_, α_2_) = (1, 1); **(H)** fused testing image using FrScatNets with (α_1_, α_2_) = (0.4, 1) and (α_1_, α_2_) = (1, 1).

The generative images on the CIFAR-10 dataset using FrScatNets with (α_1_, α_2_) = (0.4, 1) and (α_1_, α_2_) = (1, 1) are shown in Figure 4. The generative images on the CelebA dataset using FrScatNets with (α_1_, α_2_) = (1.6, 1) and (α_1_, α_2_) = (1, 1) are shown in https://mmlab.ie.cuhk.edu.hk/projects/CelebA.html.

### Image Generative Results With Image Fusion

In this subsection, we explore the impact of image fusion on the quality of the generated images using the framework of GFRSNs shown in Figure 2.

Since conventional GSNs are a good baseline for the framework of GFRSNs with different fractional orders (α_1_, α_2_), as an example, we consider the case in which the generative images from FrScatNets with different fractional orders (α_1_, α_2_), where α_1_ and α_2_ are not simultaneously equal to 1.00, are fused with the generative images from conventional ScatNets, in other words, FrScatNets with fractional orders (α_1_, α_2_) = (1, 1). Since the fractional parameters can have multiple choices, naturally, we hope to explore the effect of image fusion under different fractional parameters. All the fused images are achieved using the average method shown in Equation (17), and we choose λ = 0.5. The PSNR and SSIM results of fused images on the CIFAR-10 dataset are shown in Table 4, and those on the CelebA dataset are shown in Table 5. Note that the results are shown in the row where the “Fusion or not?” column is “Yes” in Tables 4, 5. As can be seen from the two tables, the results of PSNR and SSIM for the fused images are generally better than those for the unfused images from FrScatNets with different fractional orders (α_1_, α_2_), where α_1_ and α_2_ are not 1 at the same time. For example, when the generative images from FrScatNets with (α_1_, α_2_) = (0.4, 1) are fused with the generative images from ScatNets, the Test PSNR and Test SSIM are increased from 18.828 and 0.8514 to 18.9869 and.897, respectively, on the CIFAR-10 dataset. The results are also better than those of ScatNet-based GFRSRNs, whose Test PSNR and Test SSIM are 18.1 and 0.8352, respectively. When the generative images from FrScatNets with (α_1_, α_2_) = (1.6, 1) are fused with the generative images from ScatNets, the test PSNR and test SSIM are increased from 21.4632 and 0.9571 to 22.337 and 0.9839, respectively, on the CelebA dataset. The results are also better than those of ScatNet-based GFRSRNs, whose test PSNR and test SSIM are 21.1668 and 0.944, respectively. The fused images on the CIFAR-10 dataset are shown in Figures 4D,H and those on the CelebA dataset are shown in https://mmlab.ie.cuhk.edu.hk/projects/CelebA.html, respectively.

We also consider the generative images from FrScatNets with fractional orders (0.4, 1) and (1.6, 1), and the results are shown in the last row of Tables 4, 5, respectively. As can be seen from the two tables, the test PSNR and test SSIM are better than the fusion results of fractional orders (1.6, 1) and (1, 1) on both the CIFAR-10 and CelebA datasets.

### The Deformation Property of the Proposed GFRSNs

In this section, we evaluate the deformation property of the proposed GFRSNs as generally done in GANs. Specifically, given two images *x*_1_ and *x*_2_, we modify β to get the interpolated images:


(18) $$\begin{array}{l} x_{\beta }=G\left(\left(1-\beta \right)z_{1}+\beta z_{2}\right),\text{for}z_{1}=\Phi \left(x_{1}\right)\text{ and }z_{2}=\Phi \left(x_{2}\right), \end{array}$$

where Φ (.) denotes the fixed embedding, that is, the fractional scattering transform and Gaussianization process. The results are shown in https://mmlab.ie.cuhk.edu.hk/projects/CelebA.html.

As Angles and Mallat (2018) point out, the Lipschitz continuity to deformations of the scattering network resulting in the continuous deformation from one image to another image. https://mmlab.ie.cuhk.edu.hk/projects/CelebA.html shows that the proposed GFRSNs improve the capability to extract information while maintaining the deformation properties when compared with GSNs. On the other hand, we reproduce the morphing properties of GANs without learning a discriminator.

Besides, we should note that the generated images have strong similarities with those in the training set and, thus, lead to some unrealistic results; this is partially due to the autoencoder architecture of our model. Although under the autoencoder architecture, regarding the generative model as an inverse problem of FrScatNets, can eliminate our need to train an encoder or a discriminator, however, within this supervised paradigm, the generalization ability of the model may be limited to some extent. Therefore, when we try to recover images from unknown images, the results of the model will generate images that are similar to ones in the training set.

### Comparison Results With GANs

In this section, we compared the results of the proposed GFRSNs with GANs on the CelebA dataset.

#### Comparison Results With DCGAN and PGAN

We compare the visual results of the proposed GFRSNs with those of the DCGAN (Radford et al., 2016) and progressive GAN (PGAN)^2^ (Karras et al., 2018), as shown in https://mmlab.ie.cuhk.edu.hk/projects/CelebA.html, from which we can see that DCGAN produces a certain degree of distortion. On the contrary, the proposed GFRSNs and PGAN do not show this kind of problem. PGAN generates more image details than the proposed GFRSNs, and we think that the reasons are:

(1) The proposed GFRSNs still belong to the autoencoder architecture, which is generally inferior to that of the GANs in terms of image generation quality. However, the autoencoder has its own merits; for example, it can obtain an image code (or a latent vector), which is very helpful for downstream tasks such as image classification. In contrast, the GANs cannot generate this latent vector.
(2) The proposed GFRSNs use learning-free FrScatNets instead of CNNs in the encoder stage, which significantly reduces the parameters (for example, reducing the parameters by half compared with DCGAN). However, it also has a certain impact on image generation performance.
(3) The proposed GFRSNs can maintain the structure of the face but show smoothed results to a certain extent. The reason for this is, maybe, the choice of *L*_1_ loss.
(4) PGAN uses a more advanced low-resolution to high-resolution generation paradigm, which is more effective than the generator used in GFRSNs.

Note that we choose DCGAN as one of the compared methods, since we use the same generator architecture as the DCGAN. The reason we choose PGAN rather than the more recent BigGAN (Brock et al., 2019) as the other compared method is that the two models achieved similar results without additional class information.

#### Comparison Results With CycleGAN

We compare the objective evaluation criteria (PSNR and SSIM) with CycleGAN^3^ (Zhu et al., 2017) on the CelebA dataset. Note that SSIM and PSNR are not suitable for evaluating the quality of GANs, since GANs, generally, generate images directly from Gaussian white noise. That is, we do not have real images corresponding to the generated images, but real images are needed to calculate the PSNR and SSIM scores.

The reason we choose CycleGAN as the compared method is that it can be seen as a special kind of autoencoder model and, hence, can be used to calculate the PSNR and SSIM scores. The structure of CycleGAN is shown in https://mmlab.ie.cuhk.edu.hk/projects/CelebA.html. As in the experiment of GFRSNs, we choose 65,536 training images and 16,384 testing images. For the training process, we divide the training set into two subsets of the same size, namely, A and B, to meet the unique circular training process. By training CycleGAN through 32,768 images in domain A and 32,768 images in domain B, we can calculate Train PSNR and Train SSIM. For the testing process, we also divide the testing set into two subsets of the same size, namely, A and B, to meet the unique circular training process. By training CycleGAN through 8,192 images in domain A and 8,192 images in domain B, we can calculate Test PSNR and Test SSIM. It can be known from the experimental process that in order to calculate the PSNR and SSIM values of the training data set and the testing data set, there are several characteristics when using CycleGAN:

(1) The training and testing processes are performed separately; that is, the trained generator of CycleGAN is not used in the testing process, since CycleGAN performs the task of image-to-image translation or style transfer (Gatys et al., 2016). In order to get the Test PSNR and Test SSIM of the testing images, we still need to train CycleGAN with the testing images. For example, as is shown in https://mmlab.ie.cuhk.edu.hk/projects/CelebA.html, the generator G_AB_ takes an image from domain A, and then tries to do an image-to-image translation, so that the output will be a fake image with a style similar to domain B. However, there is only one style of images in the CelebA dataset; therefore, the generator will learn the same image as the input. That is, it is unfair to use the PSNR or SSIM score to measure the quality of CycleGAN to some extent, since CycleGAN trains the testing images.
(2) In CycleGAN, the role of the generator is not focused on generating images from noise. On the contrary, the generator takes their effort to the task of image-to-image translation. When the style of two subsets is the same, this kind of image-to-image method will undoubtedly lead to pixel-level alignment and, hence, failure of pixel error-based metrics, such as PSNR and SSIM. That is, the PSNR and SSIM scores can be seen as the upper bound of other methods.

The results of the comparison of PSNR and SSIM scores of the proposed GFRSNs with CycleGAN are shown in Table 6, from which we can see that the result of GFRSNs is worse than that of CycleGAN, especially on the testing set. This is not surprising, because CycleGAN implements style transfer between training data and testing data, while GFRSNs implements reconstruction from FrScatNet features to images. The PSNR and SSIM scores of CycleGAN can be seen as the upper bound of GFRSNs; that is, the proposed GFRSNs still have a lot of room for improvement.

**Table 6:** Quantitative results of CycleGAN and the proposed GFRSNs with fractional orders α_1_ = 0.4, α_2_ = 1 on the CelebA dataset.

	**Train PSNR**	**TestPSNR**	**Train SSIM**	**Test SSIM**
Cycle GAN	30.8059	32.6890	0.9824	0.9822
Ours	27.9721	21.4631	0.9629	0.9350

## Conclusions

This study proposes generative fractional scattering networks (GFRSNs), which use fractional wavelet scattering networks (FrScatNets) as encoder to obtain features (or FrScatNet embeddings) and deconvolutional neural networks as decoder to generate an image. Additionally, this study develops a new feature-map fusion (FMF) method to reduce the dimensionality of FrScatNet embeddings. The impact of image fusion is also discussed in this study. The experimental results on the CIFAR-10 and CelebA datasets show that the proposed GFRSNs can lead to better generated images than the original GSNs in the testing dataset. Compared with GANs, the proposed GFRSNs lack details of the generated image because of the essence of the autoencoder structure; however, the proposed GFRSNs have the following merits:

(1) They can obtain an image code (or a latent vector), which is very helpful for downstream tasks such as image classification.
(2) They use learning-free FrScatNets instead of CNNs in the encoder stage, which significantly reduces the parameters.
(3) They may have a potentially good performance in the differential privacy (DP) learning framework, since Tramer and Boneh (2021) show that ScatNet outperforms deep CNNs in differential private classifiers. We studied the image generation performance of GFRSNs under the framework of differential privacy learning. Appendix A gives some preliminary results.

## Data Availability Statement

Publicly available datasets were analyzed in this study. This data can be found here: http://www.cs.toronto.edu/~kriz/cifar.html; http://mmlab.ie.cuhk.edu.hk/projects/CelebA.html.

## Author Contributions

JW did conceptualization, methodology, writing—reviewing, and editing. XQ did validation and revised and edited the manuscript. JZ did writing—original draft preparation, software, and visualization. FW did software, validation, and data curation. YK and GY did validation and project administration. LS did formal analysis, writing—reviewing, and editing. HS did supervision and resources. All authors contributed to the article and approved the submitted version.

## Funding

This study was funded by the National Natural Science Foundation of China under Grant Nos: 61876037, 62171125, 31800825, 61871117, 61871124, 61773117, and 61872079 and in part by INSERM under the grant called IAL and IRP.

## Conflict of Interest

The authors declare that the research was conducted in the absence of any commercial or financial relationships that could be construed as a potential conflict of interest.

## Publisher's Note

All claims expressed in this article are solely those of the authors and do not necessarily represent those of their affiliated organizations, or those of the publisher, the editors and the reviewers. Any product that may be evaluated in this article, or claim that may be made by its manufacturer, is not guaranteed or endorsed by the publisher.

## Footnotes

^1^ https://github.com/tomas-angles/generative-scattering-networks

^2^ https://github.com/facebookresearch/pytorch_GAN_zoo

^3^ https://github.com/junyanz/pytorch-CycleGAN-and-pix2pix
