# Supplementary material for: Fractional Wavelet-Based Generative Scattering Networks
Source: Front Neurorobot. 2021 Oct 26;15:752752. doi: 10.3389/fnbot.2021.752752 (PMC8577828; doi:10.3389/fnbot.2021.752752)
Supplement: Supplementary file 1 [file Data_Sheet_1.docx]

Supplementary Material

# Generative fractional scattering networks under differential privacy

In this supplementary material, we give some preliminary results of GFRSNs under the framework of differential privacy (DP) learning (Dwork et al., 2006).

## Differential privacy and DP-SGD:

Differential privacy provides rigorous privacy guarantees for algorithms while allowing for privacy analysis. Specifically, a learning algorithm *A*(.) satisfies $\left( \varepsilon,\delta\right)\mathbf{-}$ differential privacy (Dwork et al, 2006), if for any datasets $D, D'$ that differ in one record, and any set of models *S*:

$$Pr\left[ A\left( D \right)\in S \right]\leq e^{\varepsilon}Pr[A(D')\in S]+\delta.$$

In our cases, mechanism *A*(.) is the training algorithm, $\varepsilon$ corresponds to the upper bound of privacy loss, and δ is the probability of breaching DP constraints. Intuitively, DP guarantees the difficulty of inferring the presence of an individual in the private dataset by observing $A\left( D \right),$ low $\varepsilon$ corresponds to low privacy lost, and hence we expect that our algorithm can maintain a good utility while keep lowering the $\varepsilon$ as much as possible.

A simple and effective approach towards the differential is replacing the typical Stochastic gradient descent (SGD) with a differentially private SGD (DP-SGD) (Abadi et al., 2016) and thereby limiting the contribution of a particular training example in the final trained model. DP-SGD enforces the desired privacy requirement by first clipping the gradients to have an L_2_-norm no larger than a predefined threshold at each training step, and then sampling random noise and adding it to the gradients, before performing gradient descent on the trained parameters. Generally speaking, the smaller the given privacy budget $\varepsilon$, the lower the utility of the DP model we trained.

## Experiments details

Throughout our experiments, we use an existing popular implementation^[[1]](#footnote-1)^ of DP-SGD. During the experiments, we set the fractional parameter$\alpha_{1}=\alpha_{1}=1$for convenience, and to make the model not so big, we set the rotational parameters $L=4$and the scattering scale $J=2$, besides, to be consistent with the existing state-of-the-art work, we evaluate our idea on the MNIST dataset and the Fashion-MNIST dataset. Note that this is a preliminary experiment, we just want to make a visual reference and a preliminary verification of GFRSNs under differential privacy, we do our experiments under $\left( {10,10}^{5} \right)$-differential privacy, and the results is shown in **Supplementary Figure 1.**

Although we just obtain the preliminary results with no hyperparameter optimization, the results of the experiments are quite satisfactory. As we can see that for the two datasets, especially Fashion-MNIST, the proposed GFRSNs reconstructs input images quite well in terms of visual effects. In other words, the GFRSNs under differential privacy is worthy of further research.

# Supplementary Figures and Tables

## Supplementary Figures


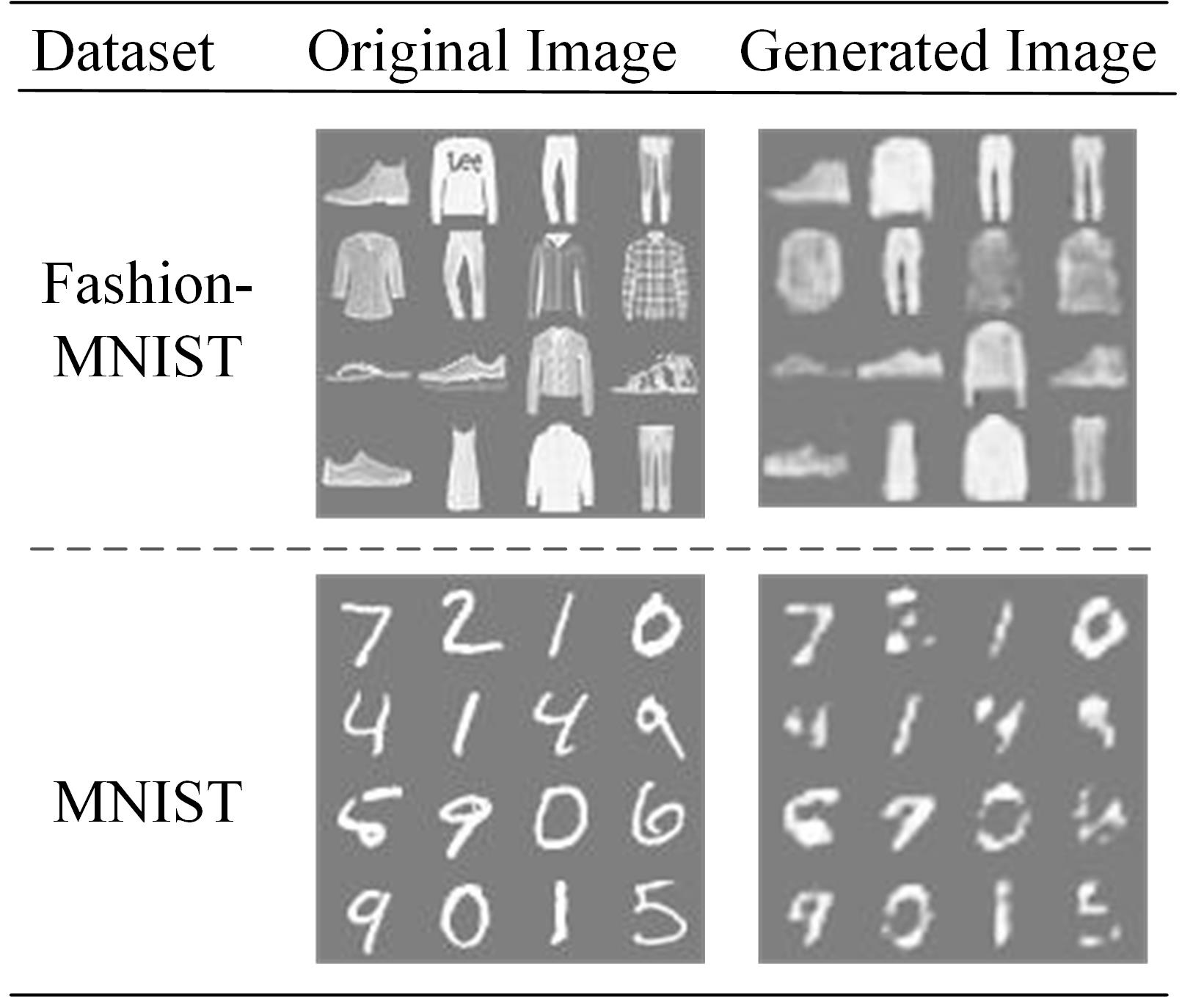


**Supplementary Figure 1.** Generated images using GFRSNs under differential privacy $(\varepsilon,\delta)=\left( {10,10}^{5} \right).$

# Reference

Abadi, M., Chu, A., Goodfellow, I., McMahan, H. B., Mironov, I., Talwar, K., and Zhang, L. (2016). Deep Learning with Differential Privacy. In Proceedings of the 2016 ACM SIGSAC Conference on Computer and Communications Security (CCS) (Vienna), 308–18. doi: 10.1145/2976749.2978318

Dwork, C., McSherry, F., Nissim, K., and Smith, A. (2006). Calibrating noise to sensitivity in private data analysis. In 2006 Proceedings of the Third Conference on Theory of Cryptography (TCC) (New York), 3876:265–84. doi: 10.1007/11681878_14

1. https://github.com/pytorch/opacus [↑](#footnote-ref-1)
